# Genome Sequencing of *Listeria monocytogenes* “Quargel” Listeriosis Outbreak Strains Reveals Two Different Strains with Distinct *In Vitro* Virulence Potential

**DOI:** 10.1371/journal.pone.0089964

**Published:** 2014-02-26

**Authors:** Kathrin Rychli, Anneliese Müller, Andreas Zaiser, Dagmar Schoder, Franz Allerberger, Martin Wagner, Stephan Schmitz-Esser

**Affiliations:** 1 Institute for Milk Hygiene, University of Veterinary Medicine Vienna, Vienna, Austria; 2 Austrian Agency for Health and Food Safety (AGES), Vienna, Austria; 3 Christian Doppler Laboratory for Molecularbiological Food Analytics, University of Veterinary Medicine, Vienna, Austria; University of Illinois at Chicago College of Medicine, United States of America

## Abstract

A large listeriosis outbreak occurred in Austria, Germany and the Czech Republic in 2009 and 2010. The outbreak was traced back to a traditional Austrian curd cheese called “Quargel” which was contaminated with two distinct serovar 1/2a *Listeria monocytogenes* strains (QOC1 and QOC2). In this study we sequenced and analysed the genomes of both outbreak strains in order to investigate the extent of genetic diversity between the two strains belonging to MLST sequence types 398 (QOC2) and 403 (QOC1). Both genomes are highly similar, but also display distinct properties: The QOC1 genome is approximately 74 kbp larger than the QOC2 genome. In addition, the strains harbour 93 (QOC1) and 45 (QOC2) genes encoding strain-specific proteins. A 21 kbp region showing highest similarity to plasmid pLMIV encoding three putative internalins is integrated in the QOC1 genome. In contrast to QOC1, strain QOC2 harbours a *vip* homologue, which encodes a LPXTG surface protein involved in cell invasion. In accordance, *in vitro* virulence assays revealed distinct differences in invasion efficiency and intracellular proliferation within different cell types. The higher virulence potential of QOC1 in non-phagocytic cells may be explained by the presence of additional internalins in the pLMIV-like region, whereas the higher invasion capability of QOC2 into phagocytic cells may be due to the presence of a *vip* homologue. In addition, both strains show differences in stress-related gene content. Strain QOC1 encodes a so-called stress survival islet 1, whereas strain QOC2 harbours a homologue of the uncharacterized *LMOf2365_0481* gene. Consistently, QOC1 shows higher resistance to acidic, alkaline and gastric stress. In conclusion, our results show that strain QOC1 and QOC2 are distinct and did not recently evolve from a common ancestor.

## Introduction

*Listeria* (*L.*) *monocytogenes* is a Gram-positive facultative intracellular food-borne pathogen, which can survive in multiple habitats like soil, vegetation, food processing plants, food, domestic and wild animals as well as humans [1]. *L. monocytogenes* has a remarkable ability to resist environmental stresses such as heavy metal ions, high salt concentration, low pH-values, low temperature, as well as low water activity [2]–[4].

In humans, mammals and birds *L. monocytogenes* can cause listeriosis, a rare but severe disease. The vast majority of listeriosis cases and outbreaks have been associated with the consumption of contaminated food, mainly dairy products, ready-to-eat deli meats and produce [5]. In recent years, a number of listeriosis outbreaks have been linked to contaminated cheese, including those made from pasteurized milk: e.g. hard cheese in Belgium 2011 (12 cases, 4 deaths) [6], Ricotta salata cheese in the USA in 2012 (22 cases, 4 deaths) [7], and Les Frères Cheese also in the USA in 2013 (6 cases, 1 death) [8]. Although *L. monocytogenes* is classified into 13 serotypes, the majority of sporadic cases and listeriosis outbreaks were caused by strains of 4b, 1/2a and 1/2b [9].

In healthy individuals listeriosis is usually restricted to a self-limiting febrile gastroenteritis, whereas in immunocompromised individuals an invasive and systemic infection can occur leading to meningitis, encephalitis and septicaemia with a high mortality rate of 25–30% [10], [11]. In addition, infection during pregnancy can lead to abortion, still-birth or septicaemia of the neonate [12].

A large listeriosis outbreak occurred in Austria, Germany and the Czech Republic in 2009/2010 due to consumption of a traditional Austrian cheese called “Quargel” [13]. Quargel is an acid curd cheese with a red smear made from skimmed pasteurized milk. Some recalled Quargel lots were highly contaminated with up to 10^6^–10^8^ colony forming units (CFU) of *L. monocytogenes* per gram of cheese [14], [15]. Molecular typing, including pulsed-field gel electrophoresis, revealed that two different *L. monocytogenes* strains, both serotype 1/2a, have been involved in this outbreak [16]. From June 2009 to January 2010 Quargel outbreak clone 1 (hereafter: QOC1) was the cause of 14 cases, including 5 with a fatal outcome, while between December 2009 and February 2010, clone 2 (hereafter: QOC2) accounted for 20 cases, which resulted in 3 fatalities. No maternal or neonatal case had been reported. The median age of the cases was 72 years (range 57 to 89) and 76% of the patients were male. Of the 34 patients, 25 were Austrian, 8 were German and one was from the Czech Republic. The underlying diseases did not differ from those generally described [17].

In recent years, genome sequencing of several *L. monocytogenes* strains have elucidated serotype- and strain-specific features of *L. monocytogenes* and have given new insights into the genetic determinants underlying virulence, pathogenicity and survival in food and food processing environments [18], [19].

Genome sequencing is a feasible tool for retrospective epidemiological analyses. Recently, Gilmour and co-authors showed that two closely related *L. monocytogenes* strains were responsible for a large food-borne listeriosis outbreak in Canada in 2008 using high-throughput genome sequencing [20]. Differences were almost exclusively due to the presence or absence of a prophage. In addition, Orsi et al. revealed that the human listeriosis outbreak in 2000 in the USA was caused by a *L. monocytogenes* strain that persisted in a food processing plant for over 12 years, and which has also been responsible for a sporadic case in 1988. Both strains had highly similar genomic backbone sequences with very few single nucleotide polymorphisms (SNPs); the main differences were found in the *comK* prophage region [21].

In this study we sequenced and analysed the genomes of the two Quargel outbreak strains QOC1 and QOC2, responsible for the multinational listeriosis outbreak in 2009/2010. Sequence comparison of the genomes enabled us to show that the two strains are related but distinct and did not evolve recently from a common ancestor. In addition, we determined the stress response of both outbreak strains and analyzed the *in vitro* virulence of strains QOC1 and QOC2 using human intestinal, hepatocytic and macrophage-like cell lines and primary mouse bone marrow-derived macrophages to investigate the correlation between genetic determinants, stress response and virulence.

## Materials and Methods

### Bacterial Strains

The two *L. monocytogenes* Quargel outbreak strains QOC1 and QOC2, both serotype 1/2a, were isolated from blood cultures of two male patients, who were hospitalized for 15 and 35 days, respectively. Both strains were isolated in Austria in January 2010 and were received from the Austrian Agency for Health and Food Safety (AGES). AGES IDs for QOC1∶930009/10, QOC2∶930014/10. The *L. monocytogenes* type strain EGDe (ATCC BAA-679) was used for comparison in cell culture virulence assays.

### DNA Isolation and Genome Sequencing

Both *L. monocytogenes* strains were cultivated under aerobic conditions at 37°C in brain heart infusion broth (BHI, Merck; with 125 rpm shaking) and harvested by centrifugation. The resulting pellet was used for DNA isolation using the QIAGEN genomic-tip columns and buffers (QIAGEN), in accordance with the recommendations of the manufacturer. Genome sequencing was performed using an Illumina GAII genome analyzer at the University of Veterinary Medicine Vienna, Austria. Sequencing was performed using paired-end sequencing technology and 101 bp read-length using Illumina standard protocols. Ten million reads were used for a *de novo* assembly using SeqManNGen (DNASTAR). For strain QOC1, the assembly of sequenced data resulted in generation of 17 contigs (>500 bp) for which there was an average coverage of 310×, while for strain QOC2, assembly of the reads resulted in 13 contigs (>500 bp), for which the average coverage was 330×. For ordering, the contigs were aligned to the *L. monocytogenes* EGDe genome using the “move contigs” option in MAUVE [22]. Some of the remaining gaps could be closed by PCR and Sanger sequencing - resulting in seven and four contigs in the final assembly for strain QOC1 and QOC2, respectively.

### Sequence Analyses

The genomes were analyzed and automatically annotated using the RAST server (http://rast.nmpdr.org ) [23] and the Microbial Genome Analysis and Annotation Platform MaGe/MicroScope (https://www.genoscope.cns.fr/agc/microscope/home) [24]. Genome comparisons and determination of homologous proteins were done using BlastP, BlastN, and tBlastN [25]. Similar to a previous study [26] we used a similarity cut-off of 60% amino acid identity and 80% coverage for identification of homologous proteins. Phylogenetic analyses of MLST genes of 13 *L. monocytogenes* strains of different serotypes were carried out using full-length MLST genes retrieved from GenBank. For each strain, the MLST genes were concatenated and aligned using Muscle implemented in MEGA5 [27]. Maximum likelihood phylogenetic trees using the Tamura-Nei model were calculated in MEGA5 with 500×resampling. All positions containing gaps and missing data were eliminated.

### Survival and Growth Experiments under Stress Conditions

Overnight cultures of QOC1 and QOC2 grown in BHI were centrifuged and adjusted to an optical density of 600 (OD_600_) of 0.1 in defined minimal medium (MM) consisting of RPMI-1640, 1% L-glutamine (both PAA, Austria) and 0.08 g/l ferric citrate (Merck) adjusted to pH 2 and pH 3 with 3% HCl and to pH 11 and pH 12 with 1 M NaOH, and synthetic gastric fluid according Cotter et al. [28]. CFU were determined by serial plating on tryptic soya agar (TSA) plates in triplicate after 2 h of incubation at 37°C. Survival (%) was calculated as CFU after 2 h of incubation under stress conditions divided by inoculated CFU multiplied by 100.

Growth of QOC1 and QOC2 under mild stress conditions (in MM adjusted to pH 5 and MM supplemented with 7.5% NaCl) was determined by measuring the OD_600_ at different time points. Each experiment was performed at least three times.

### Cell Lines

We used four different human cell lines: intestinal epithelial Caco2 (ATCC® HTB-37™), hepatocytic HepG2 (ATCC® 77400™), macrophage-like U937 (ATCC® CRL-1593.2™) and THP1 (ATCC® TIB-202™) cells. Cells were cultivated either in Eagle’s minimum essential medium (MEM, for Caco2 and HepG2) or in RPMI-1640 (for U937 and THP1) containing 2 mM L-glutamine, 10% fetal bovine serum (FBS), 100 units/ml penicillin, 100 µg/ml streptomycin sulphate and 0.25 µg/ml amphotericin B (all PAA).

In addition, primary mouse bone marrow-derived macrophages (mBMDM) were obtained by culturing mouse bone marrow in DMEM high glucose supplemented with 10% FBS (both Gibco, Invitrogen) and L cell-derived colony stimulating factor (CSF)-1 as described by Baccarini et al. [29].

### Virulence Assays

Cells were seeded in a 24-well plate at a mean cell density of 10^5^ cells per well for Caco2 and HepG2 and 10^6^ cells per well for U937, THP1 and mBMDM. U937 and THP1 were incubated for 48 h with 100 ng/ml phorbol 12-myristate 13-acetate prior the virulence assay (PMA, Sigma-Aldrich).

The i*n vitro* virulence assay was performed as recently described by Pricope et al. [30]. Briefly, bacteria were grown to the mid-logarithmic growth phase at 37°C. Cells were infected for 1 h at 37°C at a multiplicity of infection of 25. Bacterial numbers were confirmed by plating serial dilutions on TSA agar plates. The infected cells were washed with Dulbecco’s Phosphate Buffered Saline (PBS, PAA), and incubated in MEM or RPMI containing 100 µg/ml gentamicin (PAA) in order to kill extracellular bacteria. After 45 min (invasion efficiency) or 4 h (intracellular growth) the cells were lysed with 0.1% TritonX-100 (Merck). For mBMDM we used DMEM-high glucose supplemented with 3% FBS, L cell-derived CSF-1 and 50 µg/ml gentamycin. After 45 min mBMDM were either lysed to determine the invasion efficiency or media was changed to media containing 10 µg/ml gentamycin to determine the intracellular proliferation rate. Intracellular bacteria were determined by serial plating on TSA plates. CFUs were counted after incubation at 37°C for 24 h. Each experiment was done in duplicate and repeated on four separate independent occasions.

Invasion efficiency was calculated as the number of intracellular bacteria divided by the number of inoculated bacteria, multiplied by 100. The intracellular growth coefficient (IGC) was calculated as follows: IGC = (IB_t = 4 h_−IB_t = 0 h_)/IB_t = 0 h_ (IB = intracellular bacteria).

### Ethics Statement

For obtaining mBMDM, C57BL/6N (WT, wild-type) mice were purchased from Charles River Laboratories. All animal experiments were discussed and approved by the Ethics and Animal Welfare Committee of the University of Veterinary Medicine Vienna, conform to the National Authority (Austrian Federal Ministry for Science and Research; Tierversuchsgesetz – TVG ref BMWF-68.205/0243-II/3b/2011), and to the guidelines of FELASA, which match those of ARRIVE.

### Statistical Analysis

Microsoft Excel® 2007 was used for statistical analysis. Mean value and standard deviation of invasion efficiency, IGC and survival were calculated from four biological replicates, performed in duplicate for invasion efficiency and IGC, and in triplicate for survival. Values were compared statistically using two-tailed t-tests (independent variables). P-values <0.05 were considered to be significant.

### Accession Numbers

The genome sequences have been deposited in the EMBL European nucleotide archive under accession numbers CBVZ010000001 to CBVZ010000007 for *L. monocytogenes* QOC1 contigs and CBVW010000001 to CBVW010000004 for QOC2 contigs, respectively.

## Results and Discussion

Genome sequencing, assembly and subsequent gap closing resulted in a total of 7 contigs for strain QOC1, and 4 contigs for strain QOC2, respectively. With the exception of contig 7 from QOC1 all contigs were aligned successfully to the *L. monocytogenes* EGDe reference genome. Contig 7 (9498 bp) has an average coverage of 480× and is predicted to encode 14 genes; however, only five of the genes show significant matches in GenBank, where three of them have highest similarity (38 to 52% amino acid identity) to phage genes (locus_tags: *LMQOC1_70001–70014*, Table S1). Thus we cannot deduce a putative function or genomic localization for contig 7 within the QOC1 genome.

The main general features of both genomes are shown in Table 1. GC content (38.0%) and genome size are in the range found typical for *L. monocytogenes* genomes. The genome of strain QOC1 is approx. 74 kbp larger than the QOC2 genome.

**Table 1:** General features of the *L. monocytogenes* QOC1 and QOC2 genomes.

	QOC1	QOC2
Genome size (bp)*	2,931,460	2,857,445
No of contigs	7	4
G+C content (%)	38.0	38.0
No. of predicted codingsequences (CDS)	2890	2841
Average length of CDS	906	902
Coding density (%)	88.5	89.0
No. of rRNA operons	6	6
No. of tRNA genes	67	67
No. of phages#	–	–
Sequence type	403	398

*genomes are not closed.

# excluding the monocin regions and contig 7 of strain QOC1.

**Figure 1:**
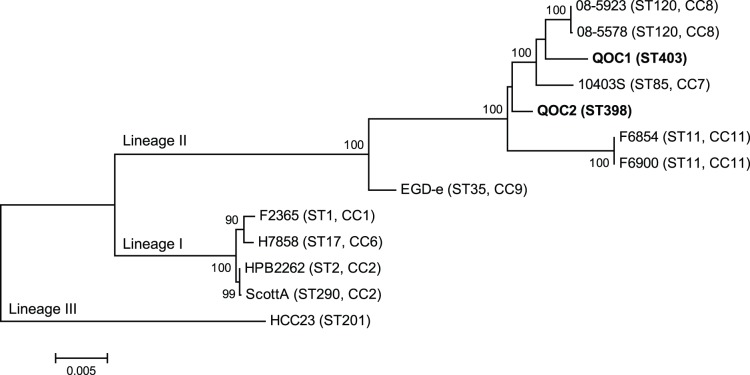
Maximum likelihood phylogenetic tree of *Listeria monocytogenes* strains based on MLST loci. *L. monocytogenes* sequence types are indicated („ST“); „CC“ denotes clonal complexes. The tree is based on concatenated full-length MLST gene sequences and was calculated with MEGA 5 [27] using the Tamura-Nei model. Bootstrap values (500× resampling) are indicated at the respective nodes.

We extracted the MLST determinants from the genome sequences according to Ragon et al. [31] and performed *in silico* MLST. Strain QOC1 belongs to sequence type (ST) 403 (encoding the following alleles: abcZ 7, bglA 7, cat 10, dapE 4, dat 5, ldh 24, lhkA 1); whereas QOC2 is a ST398 strain (abcZ 7, bglA 13, cat 19, dapE 6, dat 1, ldh 7, lhkA 1). Both strains share only two alleles. Phylogenetic analyses based on MLST genes revealed distinct grouping of the two Quargel outbreak strains within evolutionary lineage II (Figure 1). Only two other ST403 strains (both human isolates, Germany) and eight ST398 strains (from food production, environmental, and animal samples, Europe) are currently found in the *L. monocytogenes* MLST database matching with QOC1 and QOC2.

We could not find evidence for plasmids and prophages in either of the genomes, except for the monocin region (the *lma* operon) - representing a cryptic prophage – which is present in both strains and possibly also contig 7 of strain QOC1 (see above).

In *L. monocytogenes* different restriction modification (RM) systems have been shown to be involved in phage resistance [32]–[34]. Both genomes harbour a putative type II Sau3AI-like RM system, consisting of a Sau3AI-like restriction enzyme and a DNA methylase, showing 32% and 50% amino acid sequence identity to the recently described Sau3AI-like RM system of *L. monocytogenes* F2365 (serovar 4b) [32] (Figure S1). Interestingly, no homologues of the DNA binding protein *lmof2365_0326* or the recombinase *lmof2365_0328* are present in the Quargel outbreak strains (verified by blastN and tblastN searches). The Sau3AI-like RM systems, while found in the same relative genomic region (hypervariable hotspot 4), differ in their location. In QOC1 and QOC2, these systems are located between the homologues of *lmo0305* and *lmo0314*, while in F2365 and FSL N3–165, they are located between the homologues of *lmo0301* and *lmo0305*. The Sau3AI-like RM systems in QOC1 and QOC2 thus most likely represent novel Sau3AI-like RM system variants. Furthermore, the QOC2 variant harbours an additional putative helicase and nucleotidase upstream of the restriction enzyme (Figure S1). Highly similar proteins to the QOC2 RM system (99% amino acid identity) are only found in one additional *L. monocytogenes* genome: SLCC7179 (serovar 3a, ST91) isolated 1986 in Austria from cheese potentially involved in a listeriosis outbreak [35], [36]. All other homologues were from other *Firmicutes* (amino acid identity up to 63%). The GC content of the RM system genes was noticeably lower (29 to 33%) than the average *L. monocytogenes* GC content (38%), suggesting a relatively recent evolutionary horizontal gene transfer event. The presence of novel Sau3AI-like RM system variants in this particular chromosomal region emphasizes the important role of hypervariable hotspot 4 in the dissemination of RM systems among *L. monocytogenes*. No homologues of other described *L. monocytogenes* RM systems were found in QOC1 and QOC2.

Another mechanism for phage resistance are CRISPR (clustered regularly interspaced short palindromic repeats) systems [26]. Both genomes harbour one CRISPR system at locus 2 (inserted between *lmo0517* and *lmo0518* homologues), differing in their spacer content. Additionally, the CRISPR system in strain QOC2 harbours a *cas4* gene copy which is absent in QOC1.

The presence of RM and CRISPR systems might therefore be one reason for the absence of phages in the Quargel outbreak strains.

### Genes Related to Stress Resistance

*L. monocytogenes* encounters various stress conditions during its extracellular lifestyle within food, the food environment, or human hosts, as well as during intracellular replication inside eukaryotic cells. *L. monocytogenes* QOC1 encodes a homologue of the so-called “stress survival islet 1” (SSI-1), whereas strain QOC2 harbours a homologue of the (uncharacterized) *L. monocytogenes* F2365 *LMOf2365_0481* gene. SSI-1 is a 8.7 kbp region inserted between *lmo0443* and *lmo0449*, consisting of five genes (*lmo0444, lmo0445, pva, gadD1* and *gadT1*), which have been linked to tolerance towards acidic, salt, bile and gastric stress [37]–[39]. Therefore, strains harbouring SSI-1 might not only better survive in food or food production environments, but also the passage through the stomach and gut which could lead to a higher infection rate. Strains devoid of SSI-1 either encode a *LMOf2365_0481* homologue, or of the (uncharacterized) *L. innocua* genes *lin0464* and *lin0465* [40].

**Figure 2:**
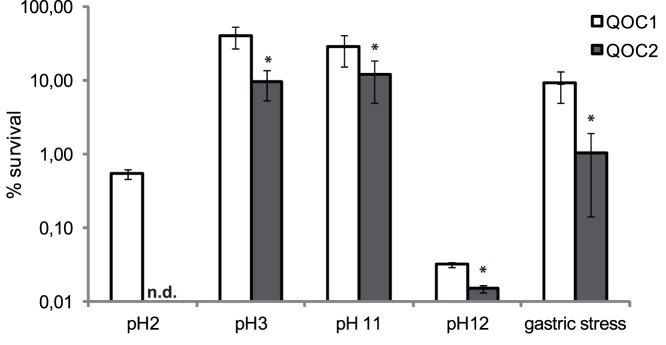
Survival of *L. monocytogenes* QOC1 and QOC2 in minimal media adjusted to pH 2, pH 3, pH 11 and pH 12; and in gastric fluid. Values, given as percentage of survival, represent mean values ± SD of four biological replicates performed in triplicate. *indicates statistical significant differences (P<0.05) between QOC1 and QOC2. n.d.: not detectable.

We therefore analyzed the stress tolerance of QOC1 and QOC2 by incubating both strains for 2 h in acid (pH 2, pH 3), alkaline (pH 11, pH 12) and in gastric fluid conditions. The survival rate of QOC1 was significantly higher under all tested conditions compared to QOC2 (Figure 2). Our results are in accordance with previous studies which demonstrate a role for SSI-1 in stress response [39]. In addition, growth of QOC1 was significantly increased under mild acidic stress (pH 5), whereas no difference was observed under salt stress (7.5% NaCl, Figure S2).

### Genome Comparison with Respect to Virulence

In general, both genomes are highly similar with respect to virulence genes, and have typical features of virulent *L. monocytogenes* genomes, such as a functional virulence gene cluster (*Listeria* pathogenicity island 1, LIPI-1), or full-length *inlAB* genes. We searched the genomes of the two Quargel outbreak strains for homologues of 81 virulence genes of *L. monocytogenes* EGDe [41]. With the exception of *lmo1099* and *lmo1102*– which are absent from both Quargel strains – and *lmo0320* (*vip*, which is missing in QOC1), homologues of all other virulence proteins were present (Table S2). Vip is an LPXTG surface protein which has been shown to be essential for the entry into host cells by recruiting Gp96 as a receptor [42], [43]. In addition, the QOC2 autolysin amidase homologue (*ami*, *lmo2558*) has a C-terminal truncation, and is thus a putative pseudogene. Ami contributes in adhesion to several eukaryotic cells and promotes colonization of hepatocytes [44], [45]. Interestingly, the QOC2 *Listeria* nuclear targeted protein A (LntA) shows only 89% amino acid and 95% nucleic acid identity to LntA from EGDe, whereas QOC1 LntA shares 99.5% amino acid identity with EGDe LntA (Table S2). LntA is secreted into the host cell nucleus and targets the chromatin repressor BAHD1 which is involved in immune response to *L. monocytogenes* infection [46]. The QOC2 LntA variant is currently found in three other sequenced *L. monocytogenes* genomes (strains: C1–387, Finland 1998, & J2–031).

Internalins and internalin-like proteins - whether secreted or located in the membrane - are essential to the virulence of *L. monocytogenes*, mainly for host cell interaction [47], [48]. Both strains encode the same set of 11 internalins (*inlA, inlB, inlC, inlC2, inlD, inlE, inlF, inlG, inlI, inlJ, inlK*). In addition, the organization of *inlAB* and *inlGC2DE* regions is identical in both strains (Figure S3, S4). Overall, strain QOC1 encodes 30 internalins or internalin-like proteins, while QOC2 encodes 26 such proteins.

**Figure 3:**
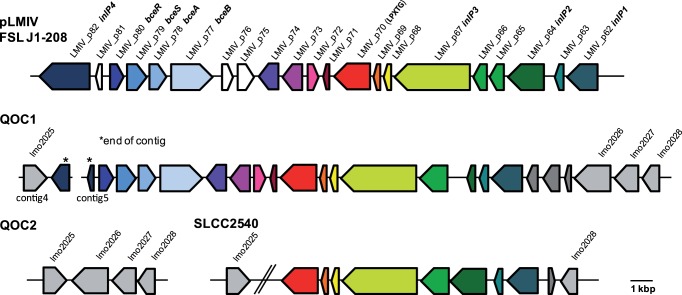
Genomic organization of the hypervariable hotspot 9 region in *L. monocytogenes* genomes harbouring homologues to proteins from plasmid pLMIV of *L. monocytogenes* FSL J1–208. Homologous proteins are shown as the same color. pLMIV and *L. monocytogenes* EGDe locus_tags are indicated.

Four putative internalins and one internalin-like protein, present only in QOC1, show highest similarity to proteins of the internalin family found on plasmid pLMIV, carried by *L. monocytogenes* strain FSL J1–208. Strain FSL J1–208, belonging to serotype 4a, was isolated from a listeriosis outbreak in goats [49]. After a more detailed analysis we identified an approx. 21 kbp region in the QOC1 genome with a GC content of 33.5% showing highest similarity to a region of pLMIV (locus_tags: LMIV_p062 to LMIV_p082; Table 2, Figure S5). This region is integrated between the *L. monocytogenes* EGDe homologues *lmo2025* (*nadA*, quinolinate synthetase) and *lmo2026* (internalin-like protein) (Figure 3), and has previously been assigned as hypervariable hotspot 9 [26]. Thus, our results strongly suggest that a part of plasmid pLMIV is integrated into the QOC1 genome, due to the significantly lower GC content of this region (33.5%) compared to the average genomic GC content (38%) and due to the high similarity to pLMIV homologues. This is most likely a relatively recent evolutionary gene transfer event.

**Table 2:** Predicted genes in the hypervariable hotspot 9 region of the *L. monocytogenes* QOC1 genome.

LMQOC1 locus_tag	Length (bp)	Description	Best Blast hit (GenBank accession no., amino acid identity)
LMQOC1_40229#	588	Internalin P4#	LMIV_p082 *L. monocytogenes* FSL J1–208 (EHY61417, 96%)
LMQOC1_50001#	150	Internalin P4#	LMIV_p082 *L. monocytogenes* FSL J1–208 (EHY61417, 90%)
LMQOC1_50002	669	Two-component response regulator bceR	LMIV_p080 *L. monocytogenes* FSL J1–208 (EHY61415, 99%)
LMQOC1_50003	333	Protein of unknown function	No hit
LMQOC1_50004	1014	Sensor histidine kinase bceS	LMIV_p079 *L. monocytogenes* pLMIV FSL J1–208 (EHY61414, 98%)
LMQOC1_50005	768	ABC transporter, ATP-binding protein bceA	LMIV_p078 *L. monocytogenes* pLMIV FSL J1–208 (EHY61413, 99%)
LMQOC1_50006	2031	ABC transporter, permease protein bceB	LMIV_p077 *L. monocytogenes* pLMIV FSL J1–208 (EHY61412, 100%)
LMQOC1_50007	876	Peptidase family M23 protein	LMIV_p074 *L. monocytogenes* pLMIV FSL J1–208 (EHY61409, 99%)
LMQOC1_50008	954	Alpha/beta hydrolase fold protein	LMIV_p073 *L. monocytogenes* pLMIV FSL J1–208 (EHY61408, 98%)
LMQOC1_50009	528	AcrR family transcriptional regulator	LMIV_p072 *L. monocytogenes* pLMIV FSL J1–208 (EHY61407, 98%)
LMQOC1_50010	153	Conserved protein of unknown function	LMIV_p071 *L. monocytogenes* pLMIV FSL J1–208 (EHY61406, 97%)
LMQOC1_50011	1746	Leucine-rich repeat domain protein(LPXTG motif)	LMOSLCC2540_2112 *L. monocytogenes* SLCC2540 (YP_006679655, 96%)
LMQOC1_50012	366	Transposase	LMIV_p069 *L. monocytogenes* pLMIV FSL J1–208 (EHY61404, 95%)
LMQOC1_50013	267	Transposase	LMIV_p068 *L. monocytogenes* pLMIV FSL J1–208 (EHY61403, 95%)
LMQOC1_50014	3612	Internalin P3	LMIV_p067 *L. monocytogenes* pLMIV FSL J1–208 (EHY61402, 94%)
LMQOC1_50015	1368	Conserved protein of unknown function	LMOSLCC2540_2116 *L. monocytogenes* SLCC2540 (YP_006679659, 96%)
LMQOC1_50016*	327	Internalin P2	LMIV_p064 *L. monocytogenes* pLMIV FSL J1–208 (EHY61399, 83%)
LMQOC1_50017	267	Transposase	LMIV_p063 *L. monocytogenes* pLMIV FSL J1–208 (EHY61398, 97%)
LMQOC1_50018	1521	Internalin P1	LMIV_p063 *L. monocytogenes* pLMIV FSL J1–208 (EHY61398, 82%)

# on two contigs.

*putative pseudogene.

The pLMIV-like region in strain QOC1 is part of two contigs in the assembly. However, the fact that the largest fragment of this region is part of contig 5 and that one part of an inlP4 homologue (*LMQOC1_40229)* is located on contig 4, strongly suggests that this region is integrated in the QOC1 genome. We performed PCR for gap closing and obtained PCR products in the expected size. In addition, sequencing of the PCR products showed >99.7% similarity to the flanking regions of contig 4 and contig 5. However, due to the high number of identical repeat units within the inlP4-like gene (*LMQOC1_40229, LMQOC1_50001*), we were unable to unambiguously close this gap (Figure S6). The pLMIV-like region in QOC1 encodes four putative internalins (inlP1 (*LMQOC1_50018*), inlP3 (*LMQOC1_50014*), inlP4 (*LMQOC1_40229, LMQOC1_50001*, whereas inlP2 is putative pseudogene), one internalin-like protein (*LMQOC1_50011*) and a small cluster encoding a putative two-component regulatory system, the ABC transporter and a few other uncharacterized proteins (Table 2). Sequence analysis revealed the highest similarity among functionally characterized proteins of the two-component regulatory system and the ABC transporter subunits (21 to 47% amino acid sequence identity) to the bceRSAB system of *Bacillus subtilis*, which is responsible for bacitracin resistance [50]. Bacitracin is a non-ribosomally synthesized peptide antibiotic produced by various bacteria. Thus, the presence of an additional putative bacitracin resistance locus in strain QOC1 may be advantageous in coping with bacterial competitors in complex microbial communities. Whether or not the presence of bceRSAB homologues in the FSL J1–208 and QOC1 genomes results in an increased bacitracin tolerance has not yet been tested. In general, most *L. monocytogenes* strains, among them QOC1 and QOC2, harbour various additional genes responsible for bacitracin resistance e.g. *telA*, *lisK* and the ABC transporter *anrB* [51], [52].

The function of the four putative internalins and the additional internalin-like protein in the pLMIV-like region is still unknown. *In vitro* virulence assays using human epithelial Caco-2 cells revealed that the invasion efficiency of the plasmid-cured *L. monocytogenes* FSL J1–208 strain was not significantly different from the parental strain, suggesting that in this experimental setting genes encoded on pLMIV are not essential for virulence [49]. However, *in vivo* virulence, the invasion efficiency and intracellular proliferation of the plasmid-cured strain FSL J1–208 have not been tested in other cell types like hepatocytes and macrophages.

More detailed sequence analysis revealed that homologues of one part of this region - including inlP1, inlP2, inlP3 and an internalin-like protein (LMIV_p070 homologue) – are also present in the hypervariable hotspot 9 region of the *L. monocytogenes* strain SLCC2540 (CLIP74906) genome, which is a serotype 3b human isolate from 1956 in the USA (Figure 3).

An additional difference between the two Quargel outbreak strains is, that only QOC2 harbours a Vip (*lmo0320*) homologue (Table S2), a surface protein covalently attached to the bacterial cell wall. Vip, which binds to the eukaryotic Gp96 surface protein, is PrfA-dependent and is essential for the entry into certain mammalian cells e.g. fibroblasts and epithelial cells. In addition, infection studies suggest a role for Vip in *Listeria* virulence, mainly in the later stages of infection [42], [43].

We also identified differences in the monocin locus, a cryptic prophage region, found in most *L. monocytogenes* genomes. In the genome of the type strain EGDe, this locus consists of the genes *lmo0115* to *lmo0129* including the *lma* operon (*lmaDCBA*) [53]. Interestingly, strain QOC2 lacks *lmaBA* homologues as well as homologues of the genes *lmo0119–0128* (Figure S7). Recently, it has been described that the genes of the *lma* operon and the surrounding prophage genes of the monocin locus are highly expressed during intracellular growth in macrophages [54]. The secreted protein LmaA provokes a delayed-type hypersensitivity response in *Listeria* immune mice, suggesting that LmaA is an immunologically relevant antigen in *Listeria* infection [55]. In addition, chromosomal deletion of *lmaB* resulted in attenuated growth of *L. monocytogenes* in the spleen and liver in a murine infection model [54]. These data suggest a role for the *lma* operon in the virulence of *L. monocytogenes* strains that harbour it. However, the presence of a truncated *lma* operon in QOC2, a feature also found in other *L. monocytogenes* strains [54], suggests that the *lma* operon is, at least in strain QOC2, not essential for virulence.

Another region with distinct differences between the two strains is the region between the *lmo0061* and *lmo0075* homologues, termed hypervariable hotspot 1 according to Kuenne et al. [26]. Interestingly, this region harbours the putative *Listeria* WSS (type VII/WXG100) secretion system. This secretion system has been shown to be important for virulence in *Staphylococcus* (*S.*) *aureus*, *Bacillus* (*B.*) *anthracis* and *Mycobacterium tuberculosis* [56]–[59], and homologues of this system have also previously been found in the *L. monocytogenes* EGDe genome [60], [61]. However, experimental evidence for the functionality as a secretion system is still missing for *L. monocytogenes* [62]. Experimental data showed that chromosomal deletion of *lmo0056* (*esxA*), a putative WXG100 protein, did not result in attenuated virulence potential in *L. monocytogenes* 10403S [63]. However, strain 10403S does not encode homologues of the whole *S. aureus* WSS region, only *esxA* to *essC* homologues are present. The putative WSS secretion system of *L. monocytogenes* 10403S might therefore be non-functional or may display different functionality compared to those putative *Listeria* WSS secretion systems harbouring *esxA* to *esaD* homologues.

The genome of strain QOC1 encodes homologues of the complete WSS region (*esxA* to *esaD*) of *S. aureus* and *B. cereus* [59] (Figure S8), whereas the genome of strain QOC2 (and those of many other *Listeria* genomes) encodes only a subset of the *S. aureus* WSS secretion system: the *esxA* to *essC* homologues. Particularly the regions downstream of *essC* (*lmo0061* homologues) and *esaD* (*lmo0066* homologues) show a high level of diversity and encode mostly uncharacterized proteins, although some proteins show weak similarities to predicted polymorphic toxin systems [64].

### *In vitro* Virulence

The two Quargel outbreak strains are highly pathogenic. Both were responsible for causing a number of reported listeriosis cases, some of which included fatal outcomes. However, based on genome sequence analyses, pronounced strain-specific differences with respect to virulence genes between QOC1 and QOC2 are evident. We therefore performed virulence assays in order to determine similarities and differences in the *in vitro* virulence potential of the two strains.

**Figure 4:**
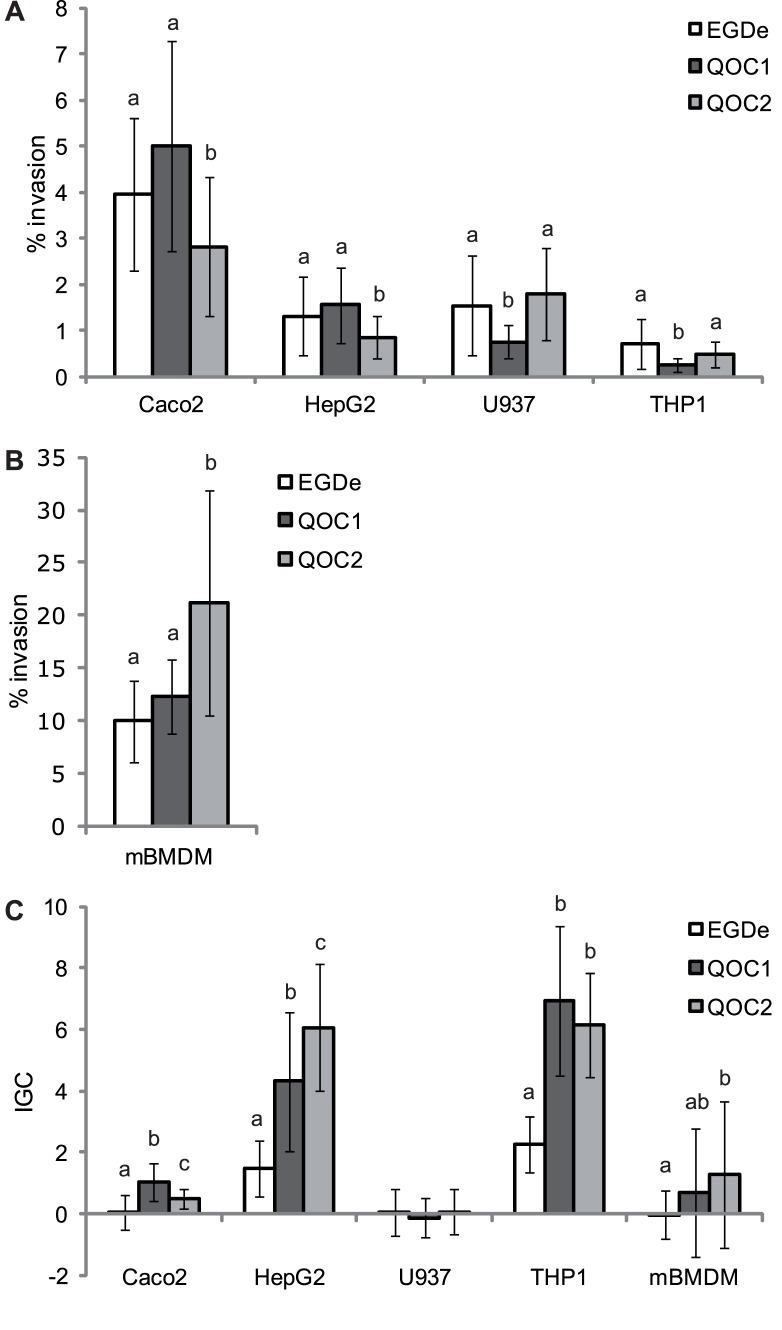
Invasion efficiency and intracellular proliferation of *L. monocytogenes* strains. Invasion efficiency (panel A and B) and intracellular growth coefficient (IGC, panel C) of type strain EGDe, QOC1 and QOC2 using four different human cell lines (intestinal epithelial Caco2, hepatocytic HepG2 and macrophage-like U937 and THP1 cells) and primary mouse bone-marrow derived macrophages (mBMDM). Values represent mean values ± SD of four biological replicates performed in duplicate. Different letters indicate statistically significant differences (P<0.05).

We evaluated invasion efficiency and intracellular growth of both Quargel outbreak strains and strain EGDe using different cell types involved in the infection pathway of *L. monocytogenes*. Invasion efficiencies of QOC1 and EGDe were significantly higher using human intestinal epithelial Caco2 and hepatocytic HepG2 cells compared to QOC2. In contrast, QOC2 showed higher invasion efficiency than QOC1 in macrophage-like cells (Figure 4). In addition, *L. monocytogenes* EGDe showed higher invasion efficiencies compared to QOC1 in U937 and THP1 cell lines. Considerable differences in intracellular growth between both Quargel outbreak strains could only be observed in non-phagocytic cells. QOC1 showed higher intracellular proliferation in Caco2, but reduced proliferation in HepG2 compared to QOC2. Interestingly, intracellular growth of EGDe in all tested host cells was low.

Our data show that both Quargel outbreak strains are virulent, however with host cell type dependent differences. The higher invasion efficiency of QOC1 observed in non- phagocytic cells could be due to the presence of the four putative additional pLMIV-like internalins, a full-length *ami* gene, the complete *lma* operon, or the complete putative *Listeria* WSS secretion system - all of which are missing or truncated in QOC2. In contrast, both QOC2 and EGDe, which show a higher number of intracellular bacteria in phagocytic cells, harbour the surface protein Vip. There is some evidence that Vip could play a role in host cell interaction between *L. monocytogenes* and macrophages due to presence of the Gp96 receptor on macrophages [65]. However, experimental data are missing. Whether or not the presence of Vip in QOC2 is responsible for increased intracellular proliferation observed in hepatocytes compared to QOC1 is unclear. A *vip* deletion mutant strain showed reduced numbers of bacteria in the liver, spleen, intestine and brain of mice [42]. The autolysin amidase Ami has been shown to promote adhesion and internalization into hepatocytic cells [44]. In line with this, QOC2, which harbors a truncated *ami* gene, shows reduced invasion into HepG2 cells compared to EGDe and QOC1 (Figure 4). However, the intracellular proliferation of QOC2 in HepG2 cells is higher compared to EGDe and QOC1, suggesting that Ami is not required for intracellular proliferation in HepG2 cells.

One explanation for the higher level of intracellular growth of QOC1 in intestinal epithelial Caco2 cells might come down to the presence of the monocin locus. Genes of the monocin locus, including the *lma* operon, which are completely present in the QOC1genome, have been described to be highly expressed during intracellular growth in macrophages [54]. Unfortunately, no data on the expression level of this locus during intracellular proliferation in other host cells like Caco2 cells are currently available.

### Comparison between Outbreak Strains

Currently, only a few genome sequences of *L. monocytogenes* strains from outbreaks with more than one strain involved are available. Orsi and coworkers analysed the genomes of four *L. monocytogenes* strains (two food and two human isolates - all serovar 1/2a) involved in a sporadic listeriosis case in 1988 (due to contaminated turkey frankfurters) and an outbreak in 2000 caused by deli turkey meat. The same food processing plant was responsible for cases in 1988 and 2000 [21]. The genomic backbone of all strains was almost identical (1 to 8 SNPs) when comparing the isolates from the same year as well as the isolates from 1988 and 2000. The only major rearrangements and variations were found in the prophage inserted into the *comK* gene. A large listeriosis outbreak occurred in Canada in 2008 due to the consumption of contaminated ready-to-eat meat. The genomes of two strains involved in this outbreak were subsequently sequenced and analysed [20]. Comparison of the two sequenced strains revealed that the genomic backbones were almost identical (28 SNPs), however, similar to the 1988–2000 listeriosis outbreak strains, the strains also differed in their prophage content: strain 08–5578 harboured a phage (φLMC1) which is absent in strain 08–5923. In addition, strain 08–5578 contained a plasmid (pLM5578), which is missing in 08–5923. Also, a comparison of four different *L. monocytogenes* strains involved in the recent cantaloupe listeriosis outbreak in the US [66], [67] using microarrays revealed strain-specific differences in phage-related genes [67]. These studies show the importance of bacteriophages in the evolution of closely related outbreak-associated *L. monocytogenes* strains. Similarly, Verghese et al. showed that phages inserted into the *comK* gene might provide fitness advantages in food production environments [68]. Unlike to the aforementioned outbreaks, no phages are found in QOC1 and QOC2. In addition, each of the Quargel strain harbours a considerable number of strain-specific genes, most of them with unknown function: *L. monocytogenes* QOC1 encodes 93 strain-specific genes, whereas strain QOC2 harbours 45 strain-specific genes (Table S1 and S3).

## Conclusion

In conclusion, our results show that strains QOC1 and QOC2 are distinct and did not recently evolve from a common ancestor. Most likely, strain QOC1 was replaced by strain QOC2, probably by changing the commercial ripening culture used for smearing [14], [15], [17]. Recent studies analysing the *L. monocytogenes* strains isolated from the Quargel lots from the Austrian producer during the last period of the outbreak (December 2009 to January 2010) revealed that all strains isolated belong to either of the two PFGE types and showed the same PFGE patterns as the human outbreak isolates. PFGE typing revealed that PFGE type 2 (the PFGE type of QOC2) was highly dominant among the food isolates from this period [69]. Similarly, QOC2 (PFGE type2) was also predominant among the human isolates in this time period [17]. Two different types of Quargel were produced in this particular plant: either red-smear-ripened, or mold-ripened. Interestingly, only the red-smear Quargel lots were *L. monocytogenes* positive [14], [69]. Furthermore, the production lines for both Quargel types were identical except for the smearing process, which was performed in different machines in physically separated areas of the production plant [14]. This strongly suggests smearing as the main contamination source with *L. monocytogenes*. Based on the available data and the pronounced genomic differences between the two *L. monocytogenes* strains involved in this outbreak, the most likely explanation is that the 2009/2010 multinational Quargel listeriosis outbreak might actually represent two overlapping outbreaks caused by separate contamination events within the same food processing plant.

## Supporting Information

Figure S1 **Genomic organization of the Sau3AI restriction system locus in** ***L. monocytogenes*** **strains of different serovar.**
(PDF)

Figure S2 **Growth under stress conditions.**
(PDF)

Figure S3 **Genomic organization of the inlAB locus in L monocytogenes serovar 1/2a outbreak strains.**
(PDF)

Figure S4 **Genomic organization of the** ***inlGHE/C2DE*** **locus in** ***L. monocytogenes*** **serovar 1/2a outbreak strains.**
(PDF)

Figure S5 **DNA-based alignment (dotplot) of pLMIV from** ***L. monocytogenes*** **FSL J1–208 with the** ***L. monocytogenes*** **QOC1 genome.**
(PDF)

Figure S6 **Genomic region surrounding the gap between contig 4 and contig 5 in the** ***L. monocytogenes*** **QOC1 genome.**
(PDF)

Figure S7 **Organization of the monocin region/** ***lmaDCBA*** **locus in** ***L. monocytogenes*** **serovar 1/2a outbreak strains.**
(PDF)

Figure S8 **Genomic organization of the putative WSS/type VII secretion system in** ***L. monocytogenes*** **serovar 1/2a outbreak strains.**
(PDF)

Table S1 **Strain-specific proteins of** ***L. monocytogenes*** **QOC1 compared with** ***L. monocytogenes*** **QOC2.**
(PDF)

Table S2 **Presence of 81 virulence-associated genes in** ***L. monocytogenes*** **Quargel outbreak strains.**
(PDF)

Table S3 **Strain-specific proteins of** ***L. monocytogenes*** **QOC2 compared with** ***L. monocytogenes*** **QOC1.**
(PDF)
