# Supplementary material for: Genome Sequencing of Listeria monocytogenes “Quargel” Listeriosis Outbreak Strains Reveals Two Different Strains with Distinct In Vitro Virulence Potential
Source: PLoS One. 2014 Feb 26;9(2):e89964. doi: 10.1371/journal.pone.0089964 (PMC3935953; doi:10.1371/journal.pone.0089964)
Supplement: Figure S1 — Genomic organization of the Sau3AI restriction system locus in L. monocytogenes strains of different serovar. (PDF) [file pone.0089964.s001.pdf]

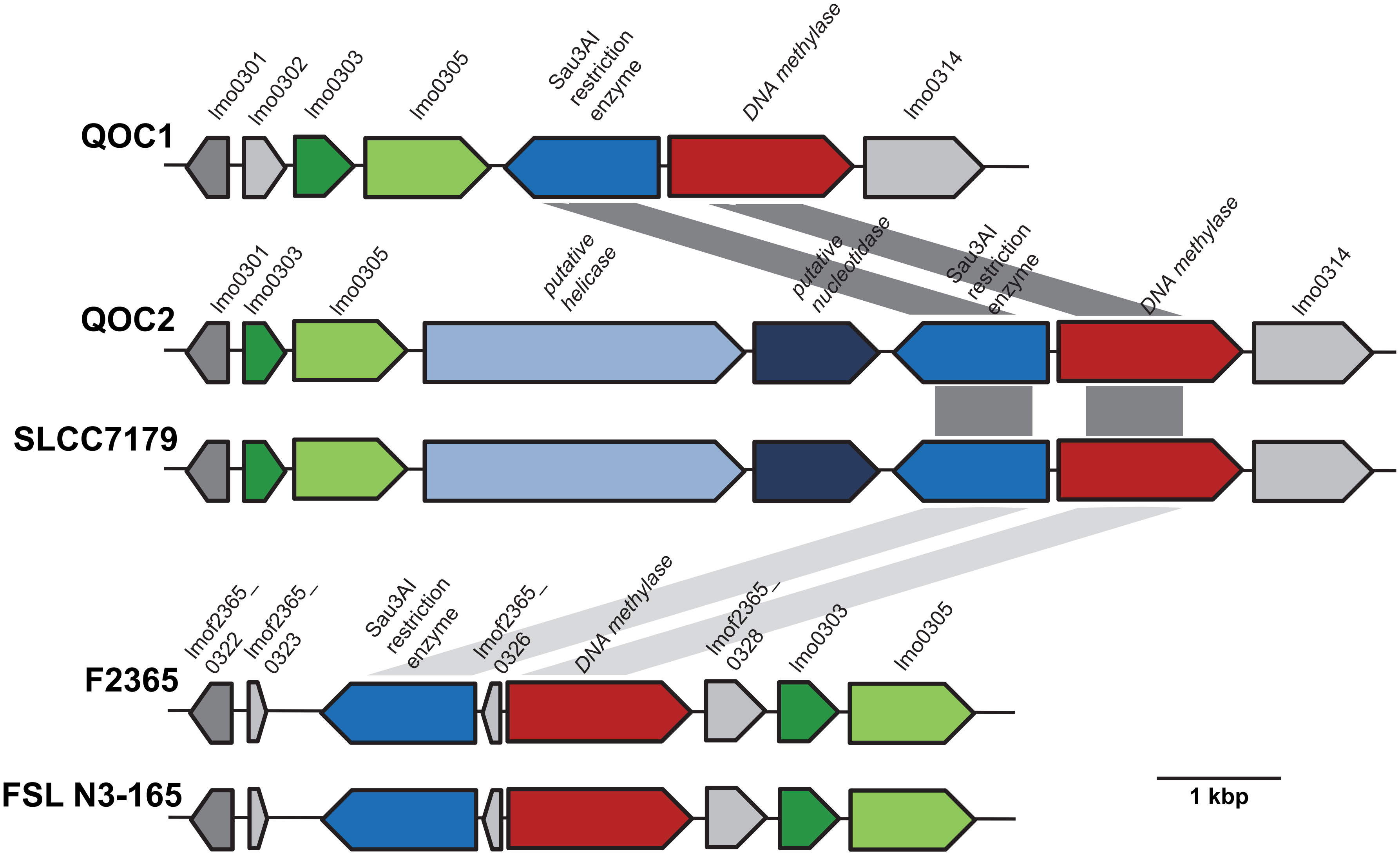

Figure S1: Genomic organization of the Sau3AI restriction system locus in *L. monocytogenes* strains of different serovar: QOC1, QOC2, FSL N3-165: serovar 1/2a; F2365: serovar 4b; SLCC7179: serovar 3a. Homologous proteins are shown in the same color. Parallelograms indicate amino acid identity between the putative restriction enzyme and the putative DNA methylase: light grey: amino acid identity below 90%, dark grey: amino acid identity above 90%. EGDe and F2365 locus_tags are indicated.
