# Supplementary material for: Genome Sequencing of Listeria monocytogenes “Quargel” Listeriosis Outbreak Strains Reveals Two Different Strains with Distinct In Vitro Virulence Potential
Source: PLoS One. 2014 Feb 26;9(2):e89964. doi: 10.1371/journal.pone.0089964 (PMC3935953; doi:10.1371/journal.pone.0089964)
Supplement: Figure S2 — Growth under stress conditions. (PDF) [file pone.0089964.s002.pdf]

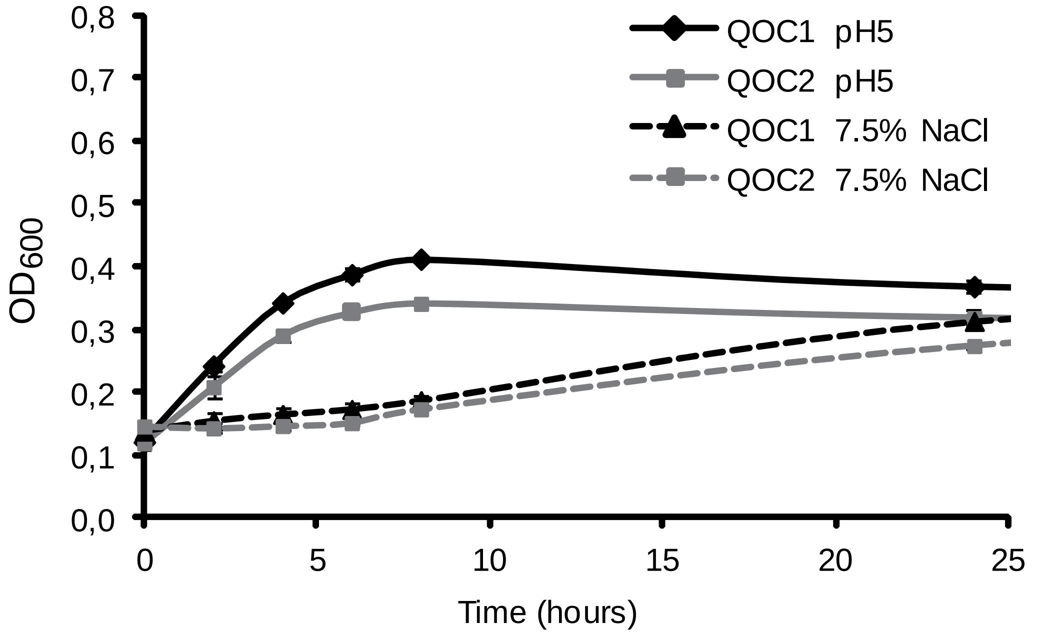

Figure S2: Growth under stress conditions

Growth of *L. monocytogenes* QOC1 and QOC2 at 37°C in minimal medium adjusted to pH5 and supplemented with 7.5% NaCl.
