# Supplementary material for: Genome Sequencing of Listeria monocytogenes “Quargel” Listeriosis Outbreak Strains Reveals Two Different Strains with Distinct In Vitro Virulence Potential
Source: PLoS One. 2014 Feb 26;9(2):e89964. doi: 10.1371/journal.pone.0089964 (PMC3935953; doi:10.1371/journal.pone.0089964)
Supplement: Figure S3 — Genomic organization of the inlAB locus in L monocytogenes serovar 1/2a outbreak strains. (PDF) [file pone.0089964.s003.pdf]

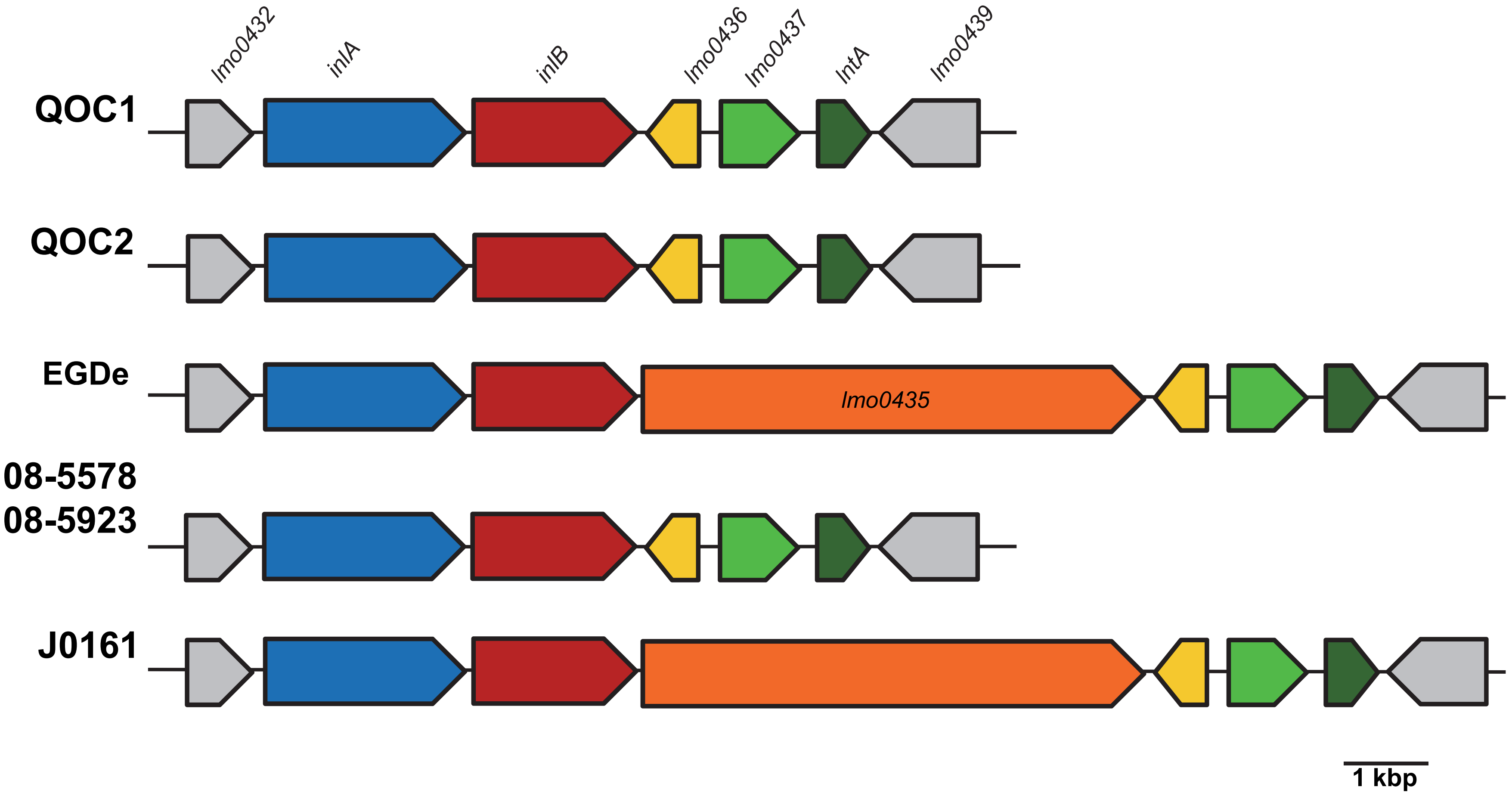

Figure S3: Genomic organization of the *inlAB* locus in *L. monocytogenes* serovar 1/2a outbreak strains. Homologous proteins are shown in the same color. *L. monocytogenes* EGDe locus_tags are indicated in the top row.
