# Supplementary material for: Genome Sequencing of Listeria monocytogenes “Quargel” Listeriosis Outbreak Strains Reveals Two Different Strains with Distinct In Vitro Virulence Potential
Source: PLoS One. 2014 Feb 26;9(2):e89964. doi: 10.1371/journal.pone.0089964 (PMC3935953; doi:10.1371/journal.pone.0089964)
Supplement: Figure S5 — DNA-based alignment (dotplot) of pLMIV from L. monocytogenes FSL J1–208 with the L. monocytogenes QOC1 genome. (PDF) [file pone.0089964.s005.pdf]

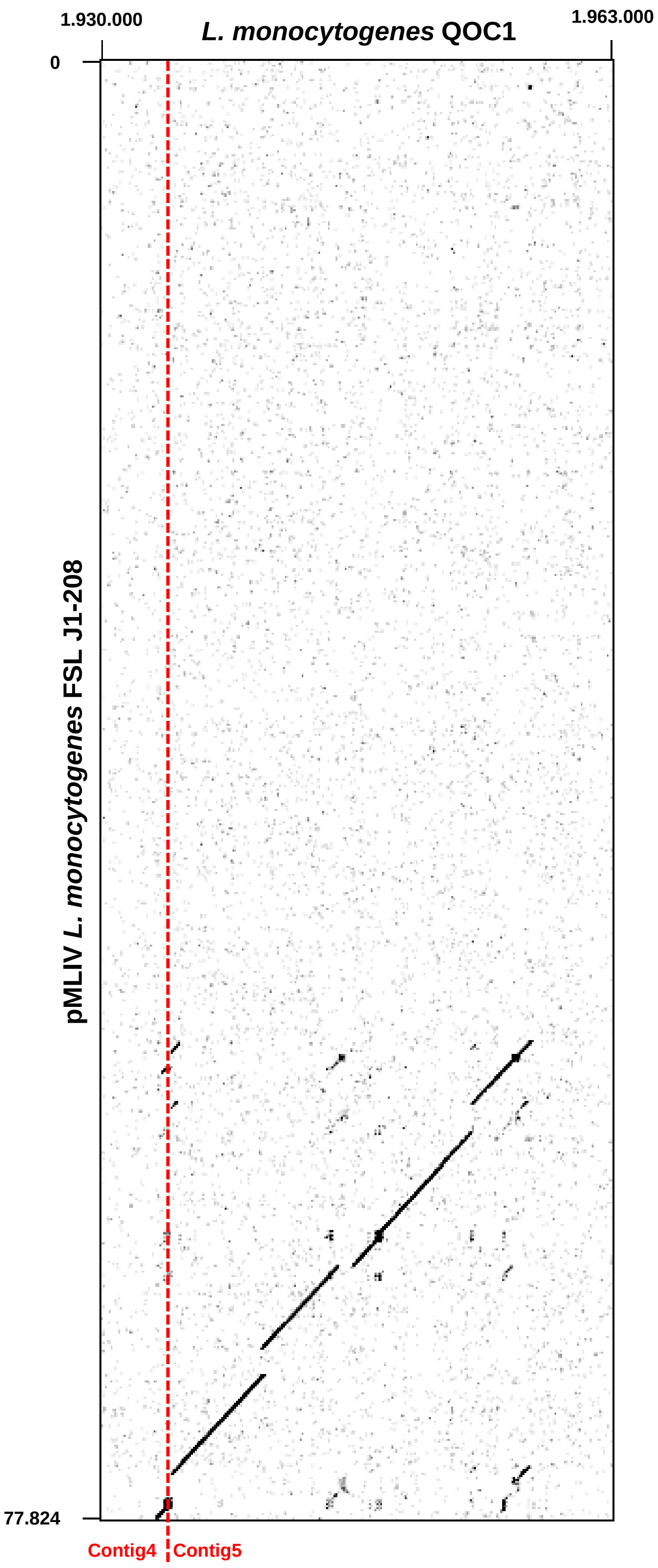

Figure S5: DNA-based alignment (dotplot) of pLMIV from *L. monocytogenes* FSL J1-208 with the *L. monocytogenes* QOC1 genome. The dotplot was created using Gepard (Krumsiek et al., Bioinformatics. 2007 Apr 15;23(8):1026-8).
