# Supplementary material for: Genome Sequencing of Listeria monocytogenes “Quargel” Listeriosis Outbreak Strains Reveals Two Different Strains with Distinct In Vitro Virulence Potential
Source: PLoS One. 2014 Feb 26;9(2):e89964. doi: 10.1371/journal.pone.0089964 (PMC3935953; doi:10.1371/journal.pone.0089964)
Supplement: Figure S6 — Genomic region surrounding the gap between contig 4 and contig 5 in the L. monocytogenes QOC1 genome. (PDF) [file pone.0089964.s006.pdf]

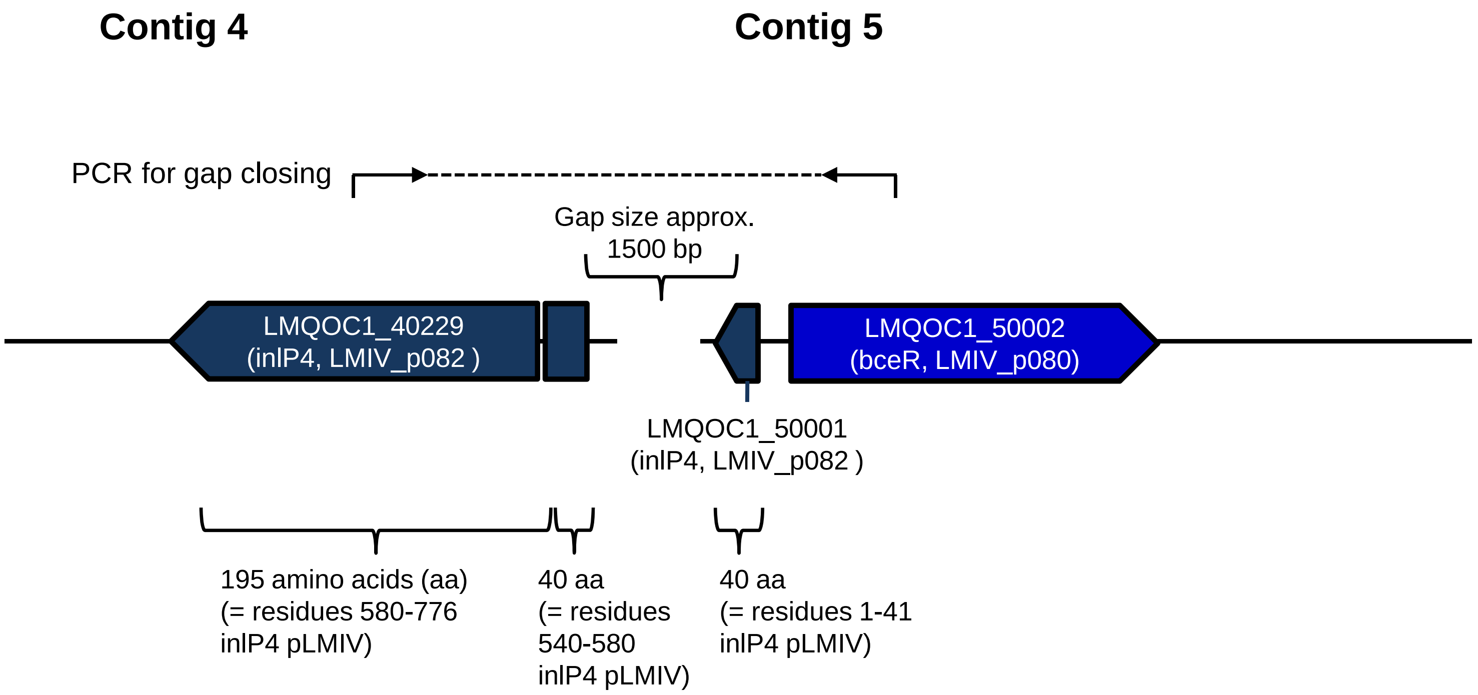

Figure S6: Genomic region surrounding the gap between contig 4 and contig 5 in the *L. monocytogenes* QOC1 genome.
