# Supplementary material for: Genome Sequencing of Listeria monocytogenes “Quargel” Listeriosis Outbreak Strains Reveals Two Different Strains with Distinct In Vitro Virulence Potential
Source: PLoS One. 2014 Feb 26;9(2):e89964. doi: 10.1371/journal.pone.0089964 (PMC3935953; doi:10.1371/journal.pone.0089964)
Supplement: Figure S7 — Organization of the monocin region/ lmaDCBA locus in L. monocytogenes serovar 1/2a outbreak strains. (PDF) [file pone.0089964.s007.pdf]

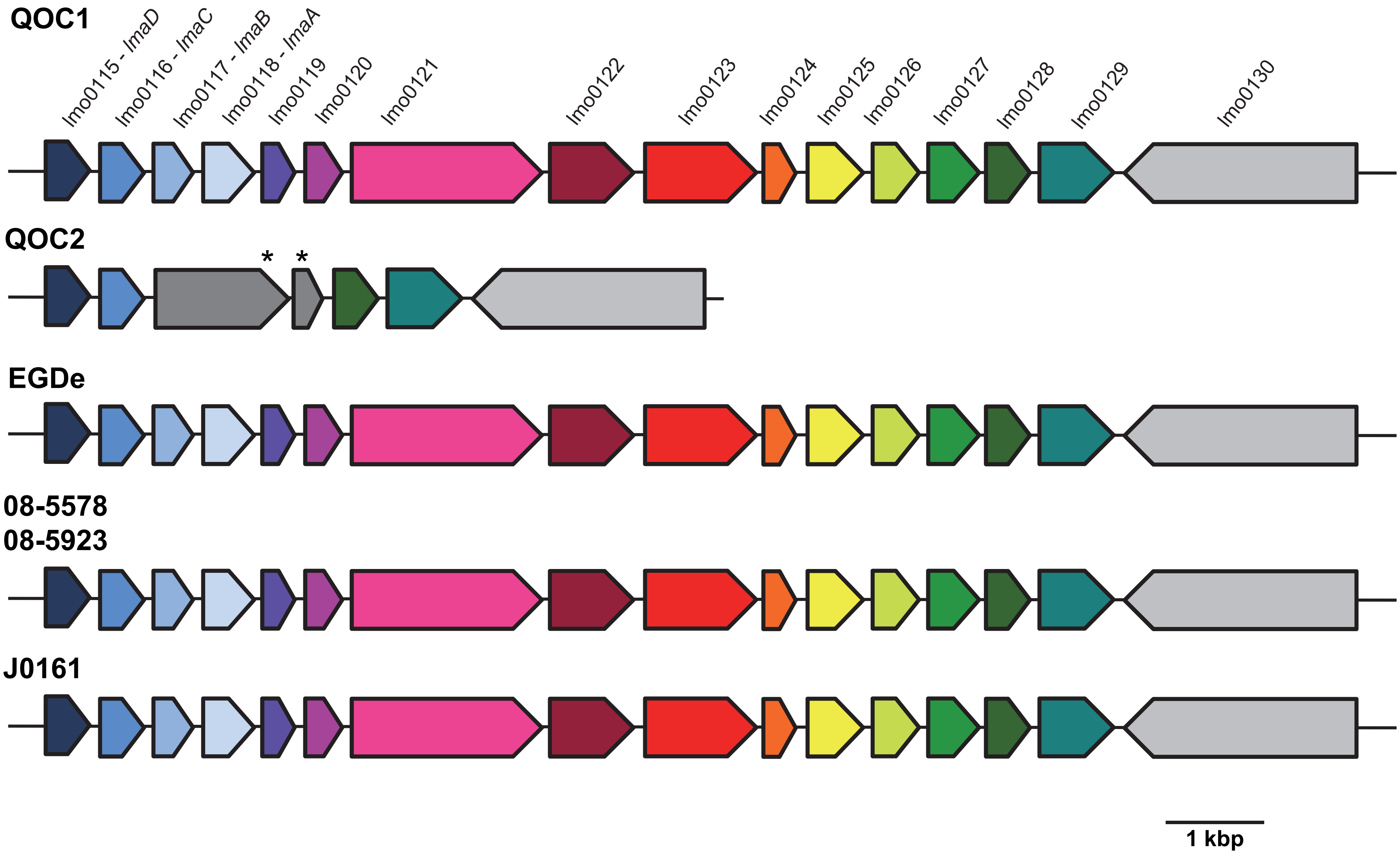

Figure S7: Organization of the monocin region/lmaDCBA locus in *L. monocytogenes* serovar 1/2a outbreak strains. Homologous proteins are shown in the same color. *L. monocytogenes* EGDe locus_tags are indicated in the top row.

*pseudogene
