# Supplementary material for: Genome Sequencing of Listeria monocytogenes “Quargel” Listeriosis Outbreak Strains Reveals Two Different Strains with Distinct In Vitro Virulence Potential
Source: PLoS One. 2014 Feb 26;9(2):e89964. doi: 10.1371/journal.pone.0089964 (PMC3935953; doi:10.1371/journal.pone.0089964)
Supplement: Figure S8 — Genomic organization of the putative WSS/type VII secretion system in L. monocytogenes serovar 1/2a outbreak strains. (PDF) [file pone.0089964.s008.pdf]

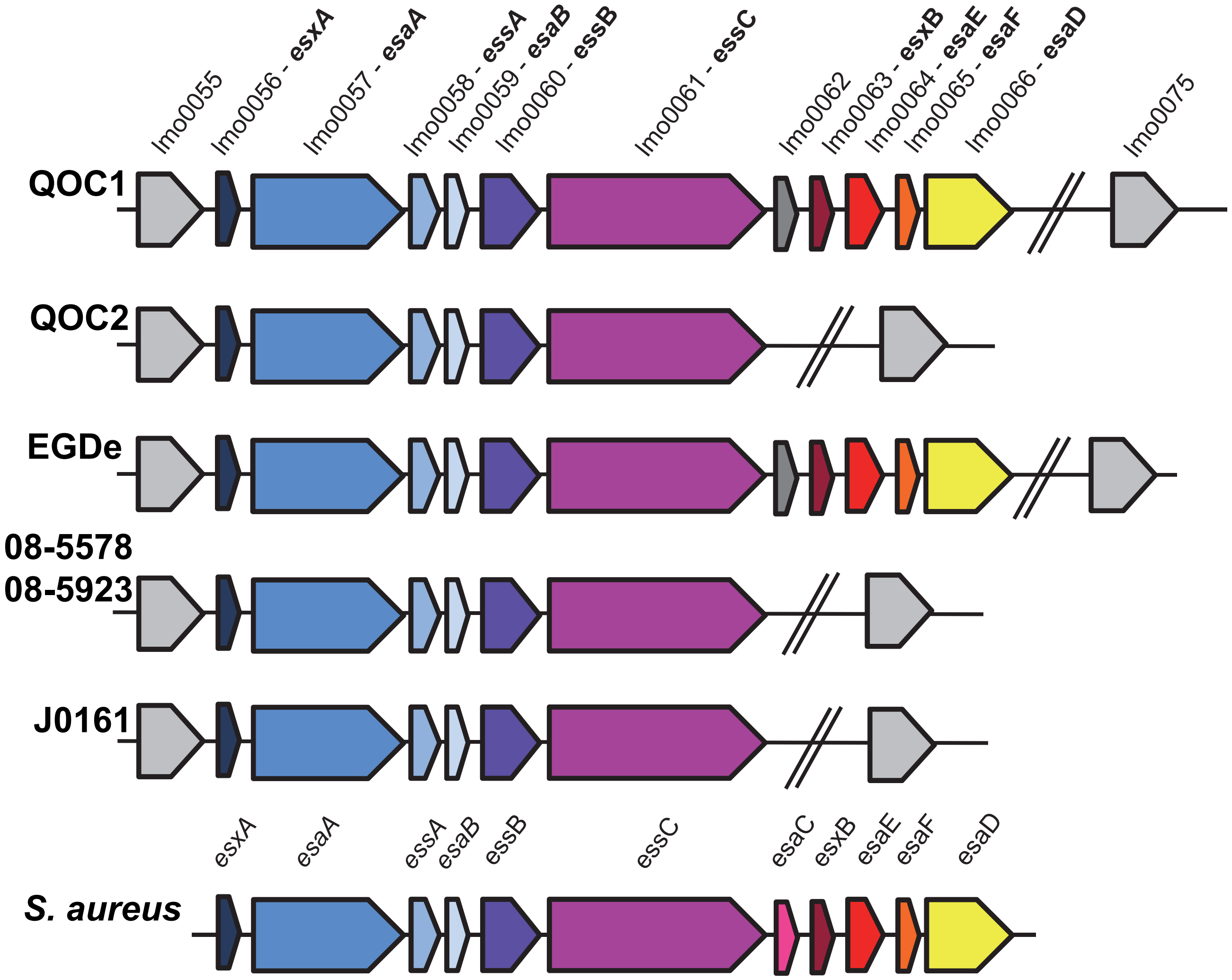

Figure S8: Genomic organization of the putative WSS/type VII secretion system
in *L. monocytogenes* serovar 1/2a outbreak strains. Homologous proteins are shown in the same color. *L. monocytogenes* EGDe locus_tags are indicated in the top row. The functionally characterized WSS/type VII secretion system of *Staphylococcus aureus* is shown at the bottom.
