# Supplementary material for: Genome Sequencing of Listeria monocytogenes “Quargel” Listeriosis Outbreak Strains Reveals Two Different Strains with Distinct In Vitro Virulence Potential
Source: PLoS One. 2014 Feb 26;9(2):e89964. doi: 10.1371/journal.pone.0089964 (PMC3935953; doi:10.1371/journal.pone.0089964)
Supplement: Table S1 — Strain-specific proteins of L. monocytogenes QOC1 compared with L. monocytogenes QOC2. (PDF) [file pone.0089964.s009.pdf]

Table S1: Strain-specific proteins of *L. monocytogenes* QOC1 compared with *L. monocytogenes* QOC2

Locus_tag	Length (bp)	Description	Best Blast hit (genbank accesion no.)	Amino acid identity to best hit
LMQOC1_20001	1590	Internalin-like protein	Internalin P4 *L. monocytogenes* FSL J1-208 (WP_003740289.1)	68%
LMQOC1_20002	555	Conserved membrane protein of unknown function	*lmo0419 L. monocytogenes* EGDe (NP_463948.1)	41%
LMQOC1_20109	3633	Hypothetical protein	*lse_0678 L. seeligeri* SLCC3954 (YP_003463917.1)	75%
LMQOC1_20145	555	Lipoprotein (Lysine rich protein)	*lmo0303 L. monocytogenes* EGDe (NP_463834.1)	99%
LMQOC1_20278	2781	Hypothetical protein	*lmo0444 L. monocytogenes* EGDe (NP_463973.1)	99%
LMQOC1_20279	1488	Hypothetical protein	*lmo0445 L. monocytogenes* EGDe (NP_463974.1)	99%
LMQOC1_20280	990	Choloylglycine hydrolase	*lmo0446 L. monocytogenes* EGDe (NP_463975.1)	99%
LMQOC1_20294	120	Hypothetical protein	*L. monocytogenes* F6900 (WP_003733942.1)	100%
LMQOC1_20295	1539	Putative transcriptional regulator	*lmo0459 L. monocytogenes* EGDe (NP_463988.1)	99%
LMQOC1_20296	2070	Conserved domain protein	*lmo0460 L. monocytogenes* EGDe (NP_463989.1)	92%
LMQOC1_20297	474	Hypothetical protein	*LMOf2365_0496 L. monocytogenes* F2365 (YP_013102.1)	99%
LMQOC1_20298	486	Hypothetical protein	*LMOSLCC2755_2081 L. monocytogenes* SLCC2755 (CBY49673)	100%
LMQOC1_20299	306	Cell wall surface anchor family protein	*L. monocytogenes* J2-064 (YP_008276409)	98%
LMQOC1_20300	267	Putative transposase orfa, IS3 family	*lmo0464 L. monocytogenes* EGDe (NP_463993.1)	98%
LMQOC1_20301	159	Hypothetical protein	*L. monocytogenes* FSL R2-561 (YP_005967382.1)	100%
LMQOC1_20302	1734	Hypothetical protein	*lmo0466 L. monocytogenes* EGDe (NP_463995.1)	98%
LMQOC1_20303	1857	Hypothetical protein	*L. seeligeri* (WP_003753995)	35%
LMQOC1_20304	1002	Hypothetical protein	*Enterococcus faecalis* (WP_002404521)	29%
LMQOC1_20305	621	Site specific recombinase/invertase, resolvase family	*LMOG_02499 L. monocytogenes* J0161 (YP_005964303)	98%
LMQOC1_20306	924	Hypothetical protein	*lse_0550 L. seeligeri* SLCC3954 (YP_003463789.1)	57%
LMQOC1_30002	156	Hypothetical protein	*lmo0473 L. monocytogenes* EGDe (YP_008475630.1)	100%
LMQOC1_30600	126	Hypothetical protein	*LMIG_02264 L. monocytogenes* FSL N3-165 (EEW14149)	100%
LMQOC1_30648	2376	Uvrd/REP helicase family protein	*L. monocytogenes* J2-064 (YP_008275427.1)	98%

LMQOC1_30649	3288	Hypothetical protein	*L. innocua* FSL J1-023 (EFR94265.1)	92%
LMQOC1_30650	294	Hypothetical protein	*Enterococcus gilvus* ATCC BAA-350 (WP_010779030)	40%
LMQOC1_30651	66	Hypothetical protein	No hits	
LMQOC1_30652	231	Hypothetical protein	UKC_00581 *Enterococcus gilvus* ATCC BAA-350 (EOI58508)	35%
LMQOC1_30653	174	Hypothetical protein	*L. monocytogenes* (WP_009929209)	68%
LMQOC1_30654	3579	Hypothetical protein	WOQ_02569 *Enterococcus faecalis* EnGen0340 (EOJ96761)	38%
LMQOC1_30655	357	Glyoxalase family protein	*LMOf2365_1111 L. monocytogenes* F2365 (YP_013711)	94%
LMQOC1_30663	438	Hypothetical protein	*LMIG_02443 L. monocytogenes* FSL N3-165 (EEW14203)	92%
LMQOC1_31198	363	Hypothetical protein	*lmo1648 L. monocytogenes* EGDe (NP_465173.1)	100%
LMQOC1_40033	1284	Hypothetical protein	*LMIG_02926 L. monocytogenes* FSL N3-165 (EEW14910)	99%
LMQOC1_40229	588	Internalin P4	*LMIV_p082 L. monocytogenes* FSL J1-208 (EHY61417)	96%
LMQOC1_50001	150	Internalin P4	*LMIV_p082 L. monocytogenes* FSL J1-208 (EHY61417)	90%
LMQOC1_50002	669	Two-component response regulator bcer	*LMIV_p080 L. monocytogenes* FSL J1-208 (EHY61415)	99%
LMQOC1_50003	333	Protein of unknown function	No hits	
LMQOC1_50004	1014	Sensor histidine kinase bces	*LMIV_p079 L. monocytogenes* pLMIV FSL J1-208 (EHY61414)	98%
LMQOC1_50005	768	ABC transporter (ATP-binding protein); efflux of cationic peptides bcea	*LMIV_p078 L. monocytogenes* pLMIV FSL J1-208 (EHY61413)	99%
LMQOC1_50006	2031	ABC transporter, permease protein bceb	*LMIV_p077 L. monocytogenes* pLMIV FSL J1-208 (EHY61412)	100%
LMQOC1_50007	876	Peptidase family M23 protein	*LMIV_p074 L. monocytogenes* pLMIV FSL J1-208 (EHY61409)	99%
LMQOC1_50008	954	Alpha/beta hydrolase fold protein	*LMIV_p073 L. monocytogenes* pLMIV FSL J1-208 (EHY61408)	98%
LMQOC1_50009	528	Acrr family transcriptional regulator	*LMIV_p072 L. monocytogenes* pLMIV FSL J1-208 (EHY61407)	98%
LMQOC1_50010	153	Conserved protein of unknown function	*LMIV_p071 L. monocytogenes* pLMIV FSL J1-208 (EHY61406)	97%
LMQOC1_50011	1746	Leucine-rich repeat domain protein (LPXTG motif)	*LMOSLCC2540_2112 L. monocytogenes* SLCC2540 (YP_006679655)	96%
LMQOC1_50012	366	Transposase	*LMIV_p069 L. monocytogenes* pLMIV FSL J1-208 (EHY61404)	95%
LMQOC1_50013	267	Transposase	*LMIV_p068 L. monocytogenes* pLMIV FSL J1-208 (EHY61403)	95%
LMQOC1_50014	3612	Internalin P3	*LMIV_p067 L. monocytogenes* pLMIV FSL J1-208 (EHY61402)	94%

LMQOC1_50015	1368	Conserved protein of unknown function	*LMOSLCC2540_2116 L. monocytogenes* SLCC2540 (YP_006679659)	96%
LMQOC1_50016*	327	Internalin P2	*LMIV_p064 L. monocytogenes* pLMIV FSL J1-208 (EHY61399)	83%
LMQOC1_50017	267	Transposase	*LMIV_p063 L. monocytogenes* pLMIV FSL J1-208 (EHY61398)	97%
LMQOC1_50018	1521	Internalin P1	LMIV_p063 *L. monocytogenes* pLMIV FSL J1-208 (EHY61398)	82%
LMQOC1_50019	555	Hypothetical protein	*lmo0419 L. monocytogenes* EGDe (NP_463948.1)	41%
LMQOC1_50020	135	Hypothetical protein	No hits	
LMQOC1_60203	2622	Putative atpase	Q9I_00731 *Enterococcus faecalis* EnGen0074 (EOE00066)	66%
LMQOC1_60530	399	Esac protein	*lmo0062 L. monocytogenes* EGDe (NP_463595)	100%
LMQOC1_60531	300	Esxb protein	*lmo0063 L. monocytogenes* EGDe (NP_463596)	100%
LMQOC1_60532	702	Esae protein	*lmo0064 L. monocytogenes* EGDe (NP_463597)	100%
LMQOC1_60533	333	Esaf protein	*lmo0065 L. monocytogenes* EGDe (NP_463598)	100%
LMQOC1_60534	1839	Esad protein	*lmo0066 L. monocytogenes* EGDe (NP_463599)	89%
LMQOC1_60535	468	Hypothetical protein	*HPL003_27375 Paenibacillus terrae* HPL-003 (YP_005078410)	58%
LMQOC1_60547	1008	Hypothetical protein	*LM5578_2950 L. monocytogenes* 08-5578 (YP_003415058.1)	98%
LMQOC1_60548	558	Putative lipoprotein	*LM5578_2949 L. monocytogenes* 08-5578 (YP_003415057.1)	99%
LMQOC1_60549	456	Hypothetical protein	*LM5578_2948 L. monocytogenes* 08-5578 (YP_003415056.1)	98%
LMQOC1_60550	426	Hypothetical protein	*LM5578_2947 L. monocytogenes* 08-5578 (YP_003415055.1)	99%
LMQOC1_60551	204	Predicted protein	*LMMG_03026 L. monocytogenes* F6900 (EEW22779)	100%
LMQOC1_60552	135	Hypothetical protein	*LMMG_03027 L. monocytogenes* F6900 (EEW22780)	100
LMQOC1_60554	729	Conserved membrane protein of unknown function	*LMMG_03027 L. monocytogenes* F6900 (EEW22787)	99%
LMQOC1_60606	390	Listeria protein lmab	*LM5578_2901 L. monocytogenes* 08-5578 (YP_003415009.1)	99%
LMQOC1_60607	513	Listeria protein lmaa	*LM5578_2900 L. monocytogenes* 08-5578 (YP_003415008.1)	100%
LMQOC1_60608	303	Hypothetical protein	*lmo0119 L. monocytogenes* EGDe (NP_463652.1)	100%
LMQOC1_60609	405	Hypothetical protein	*lmo0120 L. monocytogenes* EGDe (NP_463653.1)	99%
LMQOC1_60610	1869	Phage tail length tape-measure protein	*lmo0121 L. monocytogenes* EGDe (NP_463654.1)	99%

LMQOC1_60611	819	Phage tail fiber	*LMRG_02371 L. monocytogenes* 10403S (YP_005961270)	100%
LMQOC1_60612	1137	Minor structural protein	*LMRG_02372 L. monocytogenes* 10403S (YP_005961271)	99%
LMQOC1_60613	300	Hypothetical protein	*lmo0124 L. monocytogenes* EGDe (NP_463657.1)	100%
LMQOC1_60614	576	Hypothetical protein	*lmo0125 L. monocytogenes* EGDe (NP_463658.1)	99%
LMQOC1_60615	480	Hypothetical protein	*lmo0126 L. monocytogenes* EGDe (NP_463659.1)	100%
LMQOC1_60616	537	Hypothetical protein	*lmo0127 L. monocytogenes* EGDe (NP_463660.1)	100%
LMQOC1_70001	549	Hypothetical protein	No hits	
LMQOC1_70002	246	Hypothetical protein	P70_0013 *Listeria* phage P70 (YP_006905878)	52%
LMQOC1_70003	477	Hypothetical protein	LPLLH_ORF168 *Lactobacillus* phage LL-H (YP_001285879)	38%
LMQOC1_70004	420	Protein of unknown function	No hits	
LMQOC1_70005	342	Hypothetical protein	Head-tail connector P70_0014 *Listeria* phage P70 (YP_006905879)	51%
LMQOC1_70006	981	Hypothetical protein	*Listeria grayi* (WP_003755391)	39%
LMQOC1_70007	243	Protein of unknown function	No hits	
LMQOC1_70008	186	Protein of unknown function	*Paenibacillus elgii* (WP_010498411)	39%
LMQOC1_70009	102	Hypothetical protein	No hits	
LMQOC1_70010	303	Hypothetical protein	No hits	
LMQOC1_70011	252	Hypothetical protein	No hits	
LMQOC1_70012	276	Hypothetical protein	No hits	
LMQOC1_70013	300	Hypothetical protein	No hits	
LMQOC1_70014	174	Hypothetical protein	No hits	

* Putative pseudogene
