# Supplementary material for: Genome Sequencing of Listeria monocytogenes “Quargel” Listeriosis Outbreak Strains Reveals Two Different Strains with Distinct In Vitro Virulence Potential
Source: PLoS One. 2014 Feb 26;9(2):e89964. doi: 10.1371/journal.pone.0089964 (PMC3935953; doi:10.1371/journal.pone.0089964)
Supplement: Table S2 — Presence of 81 virulence-associated genes in L. monocytogenes Quargel outbreak strains. (PDF) [file pone.0089964.s010.pdf]

Table S2: Presence of 81 virulence-associated genes in *L. monocytogenes* Quargel outbreak strains

Gene designation	EGDe homologue	presence in *L. monocytogenes* QOC1 (% amino acid identity)	presence in *L. monocytogenes* QOC2 (% amino acid identity)
agrA	lmo0051	100,0	100,0
ctaP	lmo0135	99,6	99,6
prfA	lmo0200	100,0	100,0
plcA	lmo0201	99,1	98,4
hly	lmo0202	100,0	99,8
mpl	lmo0203	99,6	99,6
actA	lmo0204	95,3	95,3
plcB	lmo0205	99,7	99,7
lmo0206	lmo0206	98,1	98,1
ctsR	lmo0229	100,0	100,0
lmo0257	lmo0257	100,0	95,9
inlH/inlC2	lmo0263	96,4	97,5
htrA	lmo0292	100,0	100,0
vip	lmo0320	no hit	99,5
pgdA	lmo0415	99,8	99,8
inlA	lmo0433	98,4	99,0
inlB	lmo0434	99,5	99,4
lntA	lmo0438	99,5	88,8
lmo0540	lmo0540	99,8	99,8
iap	lmo0582	99,8	99,0
secA2	lmo0583	99,7	99,6
lmo0604	lmo0604	100,0	100,0
mogR	lmo0674	100,0	100,0
lmo0788	lmo0788	99,9	99,8
uhpT	lmo0838	98,1	98,7
sigB	lmo0895	100,0	100,0
lmo0915	lmo0915	100,0	100,0
lplA1	lmo0931	100,0	100,0
fri	lmo0943	100,0	100,0
dltA	lmo0974	100,0	100,0
aut	lmo1076	100,0	100,0
lmo1081	lmo1081	100,0	100,0
lmo1082	lmo1082	100,0	100,0
lmo1099	lmo1099	no hit	no hit
lmo1102	lmo1102	31,1	31,1
tig	lmo1267	100,0	100,0
sipX	lmo1269	99,5	100,0
sipZ	lmo1271	99,4	99,4
inlK	lmo1290	100,0	97,9
oat	lmo1291	99,7	99,5
hfq	lmo1295	100,0	100,0
tcsA	lmo1388	99,7	99,7

bilE	lmo1421	100,0	100,0
lmo1438	lmo1438	99,9	99,9
sod	lmo1439	100,0	100,0
lmo1521	lmo1521	100,0	100,0
relA	lmo1523	100,0	100,0
lmo1601	lmo1601	100,0	100,0
lmo1602	lmo1602	100,0	100,0
perR	lmo1683	100,0	100,0
mprF	lmo1695	100,0	99,8
adeC	lmo1742	99,7	99,7
virR	lmo1745	100,0	100,0
inlC	lmo1786	99,7	99,7
stp	lmo1821	100,0	100,0
fbpA	lmo1829	99,5	99,7
lmo1855	lmo1855	100,0	100,0
fur	lmo1956	100,0	100,0
lmo2026	lmo2026	91,2	99,7
lmo2048	lmo2048	99,4	99,4
bsh	lmo2067	99,7	100,0
lmo2114	lmo2114	100,0	100,0
lmo2115	lmo2115	100,0	100,0
dacA	lmo2120	100,0	100,0
lmo2157	lmo2157	99,8	99,7
lmo2177	lmo2177	100,0	100,0
svpA	lmo2185	100,0	99,8
oppA	lmo2196	99,8	99,8
fabF	lmo2201	100,0	100,0
lmo2203	lmo2203	99,5	99,5
clpB	lmo2206	99,9	99,9
prsA2	lmo2219	99,7	99,7
lmo2439	lmo2439	100,0	100,0
gap	lmo2459	100,0	100,0
degU	lmo2515	100,0	100,0
lmo2522	lmo2522	99,6	100,0
ami	lmo2558	99,7	95,3*
murA	lmo2691	99,3	99,7
lmo2713	lmo2713	99,7	100,0
lmo2714	lmo2714	100,0	99,7
inlJ	lmo2821	98,6	97,3

*putative pseudogene (truncated)

The list of virulence genes is based on Den Bakker et al. BMC Genomics. 2010 Dec 2;11:688.
