# Supplementary material for: Genome Sequencing of Listeria monocytogenes “Quargel” Listeriosis Outbreak Strains Reveals Two Different Strains with Distinct In Vitro Virulence Potential
Source: PLoS One. 2014 Feb 26;9(2):e89964. doi: 10.1371/journal.pone.0089964 (PMC3935953; doi:10.1371/journal.pone.0089964)
Supplement: Table S3 — Strain-specific proteins of L. monocytogenes QOC2 compared with L. monocytogenes QOC1. (PDF) [file pone.0089964.s011.pdf]

Table S3: Strain-specific proteins of *L. monocytogenes* QOC2 compared with *L. monocytogenes* QOC1

Locus_tag	Length [bp]	Description	Best Blast hit	Amino acid identity to best hit
LMQOC2_40206	360	Putative lipoprotein	*LMLG_1212 L. monocytogenes* Finland 1998 (YP_005972342)	99%
LMQOC2_40207	123	Protein of unknown function	No hits	
LMQOC2_40208	870	Putative lipoprotein	*lmo2595 L. monocytogenes* EGDe (NP_466118.1)	100%
LMQOC2_40270	1392	Putative dntp triphosphohydrolase	*LMOG_01851 L. monocytogenes* J0161 (YP_005966720)	100%
LMQOC2_40537	318	Conserved protein of unknown function	*LMRG_02492 L. monocytogenes* 10403S (YP_005961215)	99%
LMQOC2_40538	435	Conserved protein of unknown function	*LMOh7858_0077 L. monocytogenes* H7858 (EAL10133)	99%
LMQOC2_40539	309	Conserved protein of unknown function	*LMRG_02494 L. monocytogenes* 10403S (YP_005961217)	100%
LMQOC2_40542	276	Conserved protein of unknown function	*LMOh7858_0087 L. monocytogenes* H7858 (EAL10143)	100%
LMQOC2_40543	714	Conserved protein of unknown function	*LMOh7858_0088 L. monocytogenes* H7858 (EAL10144)	100%
LMQOC2_40547	408	Conserved protein of unknown function	*BN424_1238 Carnobacterium maltaromaticum* LMA28 (CCO10735)	49%
LMQOC2_40550	294	Conserved protein of unknown function	*lmo0069 L. monocytogenes* EGDe (NP_463602)	98%
LMQOC2_40603*	204	Protein of unknown function	LMHCC_2507 *L. monocytogenes* HCC23 (ACK40842))	100%
LMQOC2_40604*	1077	Protein of unknown function	LMHCC_2507 *L. monocytogenes* HCC23 (ACK40842))	99%
LMQOC2_20106	387	Transcriptional repressor, blai/meci family	*lmo0252 L. monocytogenes* EGDe (NP_463783.1)	99%
LMQOC2_20107	1041	Regulatory sensor-transducer, blar1/mecr1 family	*lmo0253 L. monocytogenes* EGDe (NP_463784.1)	99%
LMQOC2_20108	663	Hypothetical protein	*lmo0254 L. monocytogenes* EGDe (NP_463785.1)	100%
LMQOC2_20128	2955	Hypothetical protein, putative ntpase (NACHT family)	*LMOSLCC2376_0245 L. monocytogenes* SLCC2376 (YP_006689443.1)	98%
LMQOC2_20152	1407	Conserved hypothetical protein, AAA-type atpase	*UKC_01057 Enterococcus gilvus* ATCC BAA-350 (EOI56872.1)	37%
LMQOC2_20153	519	Protein of unknown function	*HMPREF9282_00162 Veillonella ratti* ACS-216-V-Col6b (EKU79354.1)	23%
LMQOC2_20154	876	Conserved hypothetical protein protein	*HPL003_07175 Paenibacillus terrae* HPL-003 (YP_005074421.1)	27%
LMQOC2_20155	126	Hypothetical protein	*HMPREF9519_01901 Enterococcus faecalis* TX1346 (EFU17174.1)	71%
LMQOC2_20156	1239	Hypothetical protein	*lse_0277 L. seeligeri* SLCC3954 (YP_003463516.1)	59%
LMQOC2_20157	810	Hypothetical protein	*HMPREF9505_00770 Enterococcus faecalis* TX0109 (EFM70909.1)	39%
LMQOC2_20165	2652	Putative DNA/RNA helicase	*LMOSLCC7179_0296 L. monocytogenes* SLCC7179 (YP_006697989.1)	99%
LMQOC2_20166	933	5'-nucleotidase	*LMFG_00788 L. monocytogenes* FSL J2-071 (EFD91373.1|)	100%

LMQOC2_20176	1200	Cell wall surface anchor family protein	*lmo0320 (vip) L. monocytogenes* EGDe (NP_463850.1)	99%
LMQOC2_20229	1188	Membrane protein of unknown function	*Enterococcus faecalis* (WP_010709719.1)	30%
LMQOC2_20238	456	Hypothetical protein	*LMRG_00071 L. monocytogenes* 10403S (AEO05392.1)	99%
LMQOC2_20302	549	Hypothetical protein	*LMOSLCC7179_0432 L. monocytogenes* SLCC7179 (CBY59638.1)	99%
LMQOC2_20314	210	Hypothetical protein	*LMRG_02887 L. monocytogenes* 10403S (AEO05470.1)	100%
LMQOC2_20315	546	Hypothetical protein	*LMRG_00151 L. monocytogenes* 10403S (AEO05471.1)	99%
LMQOC2_20366	489	CRISPR-associated recb family exonuclease Cas4a	*M642_15185 L. monocytogenes* C1-387 (AGR03388)	99%
LMQOC2_20468	534	Acetyltransferase, GNAT family	*lmo0614 L. monocytogenes* EGDe (NP_464141.1)	99%
LMQOC2_20645	1974	Cell wall surface anchor family protein (LPXTG motif)	*LMOf6854_0833 L. monocytogenes* F6854 (WP_003723802.1)	99%
LMQOC2_20962	678	Hypothetical protein, putative transcriptional regulator	*LMOSLCC2376_1068 L. monocytogenes* SLCC2376 (CBY63100.1)	96%
LMQOC2_20963	702	Hypothetical protein, putative DNA binding protein	*LMOSLCC2376_1069 L. monocytogenes* SLCC2376 (CBY63101.1)	96%
LMQOC2_20964	810	Hypothetical protein	*LMBG_00132 L. monocytogenes* FSL J1-194 (EFG00615)	99%
LMQOC2_20971	504	Hypothetical protein	*LMHCC_1530 L. monocytogenes* HCC23 (YP_002350488.1)	99%
LMQOC2_20972	306	Hypothetical protein	*LMOf6854_1160 L. monocytogenes* F6854 (EAL07611.1)	100%
LMQOC2_20973	474	Hypothetical protein	*LMOf6854_1159 L. monocytogenes* F6854 (EAL07501)	97%
LMQOC2_20974	426	Hypothetical protein	*LMOf6854_1161 L. monocytogenes* F6854 (EAL07502)	96%
LMQOC2_21040	153	Hypothetical protein	*HMPREF0557_01844 L. innocua* ATCC 33091 (EHN61104.1)	76%
LMQOC2_30208	105	Hypothetical protein	*LMOSLCC2372_2071 L. monocytogenes* SLCC2372 (YP_006685398.1)	97%
LMQOC2_30306	441	Hypothetical protein	*LMRG_01254 L. monocytogenes* 10403S (YP_005963216)	100%
LMQOC2_30307	672	Hypothetical protein	*LMRG_01255 L. monocytogenes* 10403S (YP_005963217)	100%

* putative pseudogenes, probable split gene
